# Machine learning and population pharmacokinetics: a hybrid approach for optimizing vancomycin therapy in sepsis patients

**DOI:** 10.1128/spectrum.00499-25

**Published:** 2025-03-31

**Authors:** Keyu Chen, Chuhui Wang, Yu Wei, Sinan Ma, Weijia Huang, Yalin Dong, Yan Wang

**Affiliations:** 1 Department of Pharmacy, The First Affiliated Hospital of Xi’an Jiaotong University, Xi’an, China; 2 Department of Pharmacy, The Second Affiliated Hospital of Xi’an Jiaotong University, Xi’an, China; Innovations Therapeutiques et Resistances (INTHERES), Toulouse, France

**Keywords:** population pharmacokinetics, machine learning, sepsis, vancomycin, drug exposure

## Abstract

Predicting vancomycin exposure is essential for optimizing dosing regimens in sepsis patients. While population pharmacokinetic (PPK) models are commonly used, their performance is limited. Machine learning (ML) models offer advantages over PPK models, but it remains unclear which model—PPK, Bayesian, ML, or hybrid PPK-ML—is best for predicting vancomycin exposure across different clinical scenarios in sepsis patients. This study compares the performance of these models in predicting the 24 hour area under the blood concentration curve (AUC_24_) to support precision dosing in sepsis care. Data from sepsis patients treated with intravenous vancomycin were sourced from the MIMIC-IV database. The data set was split into training and testing sets, and four models—PPK, Bayesian, ML, and hybrid—were developed. In the testing set, AUC_24_ was predicted using all models, and performance was evaluated using mean absolute error, mean squared error, root mean squared error, mean absolute percentage error (MAPE), and R². A total of 4,059 patients were included. In the absence of vancomycin concentration data, the hybrid model outperformed both PPK and Bayesian models, with MAPE improvements of 58% and 17%, respectively. When vancomycin concentration data were available, the Bayesian model demonstrated the best performance (MAPE: 13.37% vs 68.17%, 34.17%, and 28.52% for PPK, Random Forest, and hybrid models). The hybrid model is recommended to predict AUC_24_ when concentration data were unavailable, while the Bayesian model should be used when concentrations were available, offering robust strategies for precise vancomycin dosing in sepsis patients.

**IMPORTANCE:**

This study evaluates and compares the performance of four models—PPK, Bayesian, ML, and hybrid PPK-ML—in predicting vancomycin exposure (AUC_24_) in sepsis patients using real-world data from the MIMIC-IV database. These results underscore the importance of selecting appropriate models based on the availability of concentration data, providing valuable guidance for precision dosing strategies in sepsis care. This work contributes to advancing personalized vancomycin therapy, optimizing dosing regimens, and improving clinical outcomes in sepsis patients.

## INTRODUCTION

Infections are prevalent among patients in the intensive care unit (ICU), frequently progressing to sepsis, which is associated with poor prognoses and, in many cases, death. Recent studies have highlighted an increasing frequency of vancomycin use in the ICU for the treatment of sepsis (1). Vancomycin is characterized by a narrow therapeutic index (2) and significant interindividual variability (3). Therefore, determining the appropriate vancomycin exposure is crucial for treating sepsis patients (4), with the goal of improving therapeutic outcomes and minimizing adverse reactions (5). Currently, the population pharmacokinetics (PPK) model is widely used to obtain vancomycin exposure of patients (6). Previous studies have shown that targeting the area under the blood concentration curve (AUC) is more effective than using concentration for vancomycin therapy (7). The PPK model is frequently combined with the Bayesian method to account for both interindividual and intraindividual variability. This approach can calculate 24 hour AUC (AUC_24_) of antibiotics and assist in dose adjustment in clinical practice (8). However, there are limitations in using the PPK or Bayesian models to predict AUC_24_ in clinical settings (9). For example, most of the PPK models rely on sparse sampling, leading to suboptimal prediction accuracy. Additionally, obtaining vancomycin concentrations is a standard practice in most ICU settings with available resources. However, in resource-limited areas, such as low- and middle-income countries or some special circumstances, obtaining these concentrations can still present challenges (2).

With the development of artificial intelligence technology, machine learning (ML) has garnered increasing attention. ML is a data-driven approach that uses training data to learn tasks through various algorithms, enabling decision-making and predictions for specific events (10). However, since ML models are primarily driven by correlations, false correlations present a significant risk to their generalizability. Therefore, addressing the influence of confounding variables is crucial. Additionally, ML often struggles to provide transparent explanations for the rationale behind its predictions (11). In contrast, PPK models combine classical pharmacokinetic principles with statistical models, providing higher interpretability due to their mechanism-based mathematical framework (2). A hybrid model integrating the PPK model and ML model is expected to enhance the predictive accuracy of PPK models, improve the interpretability of ML models, and overcome the limitations of Bayesian models, which rely on concentration data for predictions.

Existing studies have shown that ML-PPK hybrid models outperform both standalone PPK and ML models (12, 13). However, some research suggested that the predictive performance of ML models is comparable to or slightly lower than that of Bayesian models (14). No studies have compared the performance of the PPK model, Bayesian model, ML model, and hybrid model in predicting AUC_24_ of vancomycin in sepsis patients. Therefore, this study aims to evaluate the predictive performance of these four models for AUC_24_ in critically ill sepsis patients receiving intravenous vancomycin, with the goal of guiding individualized drug dosing.

## MATERIALS AND METHODS

### Data collection

The research data were sourced from the publicly available MIMIC-IV database, which contains electronic health records from the Beth Israel Deaconess Medical Center. The database includes information such as patient measurements, orders, diagnoses, procedures, treatments, and de-identified free-text clinical notes (15). Patient data were extracted from MIMIC-IV 2.2, with the inclusion criteria as follows: patients admitted to the ICU and diagnosed with sepsis according to the Sepsis-3 (16), patients aged over 18 years at ICU admission, patients with documented intravenous vancomycin use during their ICU stay, patients who stayed in the ICU for more than 24 hours, and patients with vancomycin concentration measurements. The exclusion criteria were as follows: patients receiving renal replacement therapy, pregnant patients, patients without dosing information prior to concentration measurements, and patients who died within 48 hours. The collected data included baseline patient information such as age, weight, and sex; laboratory data, including creatinine, creatinine clearance, and red blood cell count; vancomycin treatment regimen and blood concentrations; and hospitalization-related information such as length of stay.

### Data processing

Variables with more than 20% missing data were excluded. For the remaining variables, missing values were imputed using the MICE package in R, employing the classification and regression trees method (17). Patients with both peak and trough concentration measurements within the same dosing interval were designated as the testing set (where peak concentration was defined as the concentration measured 1–2 hours after dosing, and trough concentration was measured 30 minutes to 1 hour before the next dose), while the remaining patients were used to train the PPK model, Bayesian model, ML model, and hybrid model as the training set.

### Population pharmacokinetic model

The PPK model was developed using the nonlinear mixed-effects modeling approach (NONMEM, version 7.5; ICON Development Solutions, MD, USA). Structural models were selected from both one-compartment and two-compartment models with first-order elimination.

Vancomycin pharmacokinetic parameters demonstrate variability at both the interindividual and intraindividual levels. The interindividual random effects were represented using an exponential model:


(1) $$\begin{array}{c} P_{ij}=TV\left(P_{j}\right)*e^{\eta^{ij}} \end{array}$$

In the model, P_ij_ represents the pharmacokinetic parameter for the i-th individual and the j-th parameter. TV(P_j_) denotes the typical population value for the j-th pharmacokinetic parameter. η^ij^ represents the individual random error for the parameter P_ij_ relative to the population typical value TV(P_j_). This error was assumed to follow a normal distribution with a mean of 0 and variance ω^2^.

The residual variability model was used to describe variability beyond interindividual variability. The following models were considered for selection:


(2) $$\begin{array}{c} Additive\;error\;model:\;C_{obs}=C_{pred}+\varepsilon \end{array}$$

(3) $$\begin{array}{c} Proportional\;error\;model:C_{obs}=C_{pred}*\left(1+\varepsilon \right) \end{array}$$

(4) $$\begin{array}{c} Combined\;error\;model:C_{obs}=C_{pred}*\left(1+\varepsilon \right)+\varepsilon_{1} \end{array}$$

(5) $$\begin{array}{c} Exponential\;error\;model:C_{obs}=C_{pred}*exp^{\varepsilon } \end{array}$$

In the above equations, C_obs_ and C_pred_ represent the measured and predicted concentrations, respectively, and the residual error ε represents the model error caused by unknown factors. ε and ε_1_ fit a normal distribution centered at 0 with variance δ^2^ and δ_1_^2^.

After establishing the basic model, the stepwise regression method was used to screen covariates to establish the final PPK model. The model’s validity was assessed using a goodness-of-fit diagnostic chart and the bootstrap method.

The final PPK model was then applied to the testing set to predict C_max_ and C_min_ concentrations under two scenarios: first, using the PPK model to predict C_max_ and C_min_ in the absence of vancomycin concentration data, and second, applying a Bayesian model when vancomycin concentrations were available.

### Machine learning model

Two models were developed: an ML model and a hybrid model combining ML with the PPK model. The primary objective of both models was to predict vancomycin AUC_24_. However, due to the sparse sampling in the training data set, direct calculation of AUC_24_ was unfeasible. Consequently, concentration was chosen as the initial prediction target for both models.

The mlr3 package was employed to benchmark eight ML algorithms on the training data set aiming to identify the two with the best predictive performance. The models evaluated included GLM with elastic net regularization, k-nearest neighbors regression, linear model, neural network, random forest, decision tree, support vector machine, and XGBoost (18). The ML model incorporated all extracted variables, while the hybrid model additionally included the individual clearance (CL) and apparent volume of distribution (V) of patients. Both models underwent 10-fold cross-validation on the training set. The most suitable ML model for this study was selected on mean absolute error (MAE), mean squared error (MSE), root mean squared error (RMSE), and R^2^ scores of each model in the internal testing set.

The SHAP method (19) was employed to generate a swarm plot illustrating the contribution of each feature to the prediction results, while the Boruta method (20) was combined with clinical expertise for variable selection. To enhance model reliability and stability, hyperparameter tuning was performed, resulting in the final ML and hybrid models. These optimized models were then applied to the testing set to predict concentrations for each patient.

### Calculation of exposure

This study involved converting model-predicted concentration data to AUC_24_. AUC_24_ was calculated using the modified trapezoidal method (21), as shown in the following equation:


(6) $$\begin{array}{c} AUC_{24}=\left(\frac{t_{inf}\times \left(C_{max}+C_{min}\right)}{2}+\frac{\left(C_{max}-C_{min}\right)\times \Delta t}{\ln C_{max}-\ln C_{min}}\right)\times n \end{array}$$

C_max_ is the measured peak concentration, C_min_ is the measured trough concentration, ∆t is the time difference in hours between the two concentrations, t_inf_ is the vancomycin infusion time in hours, and *n* is the number of doses administered in 24 hours.

By substituting the true concentrations of patients in the testing set into equation 6, the true value of AUC_24_ was obtained. Equation 6, along with the C_max_ and C_min_ values derived from the four models in the testing set, was used to calculate the predicted AUC_24_ for each patient.

### Model performance comparison

**Fig 1:**
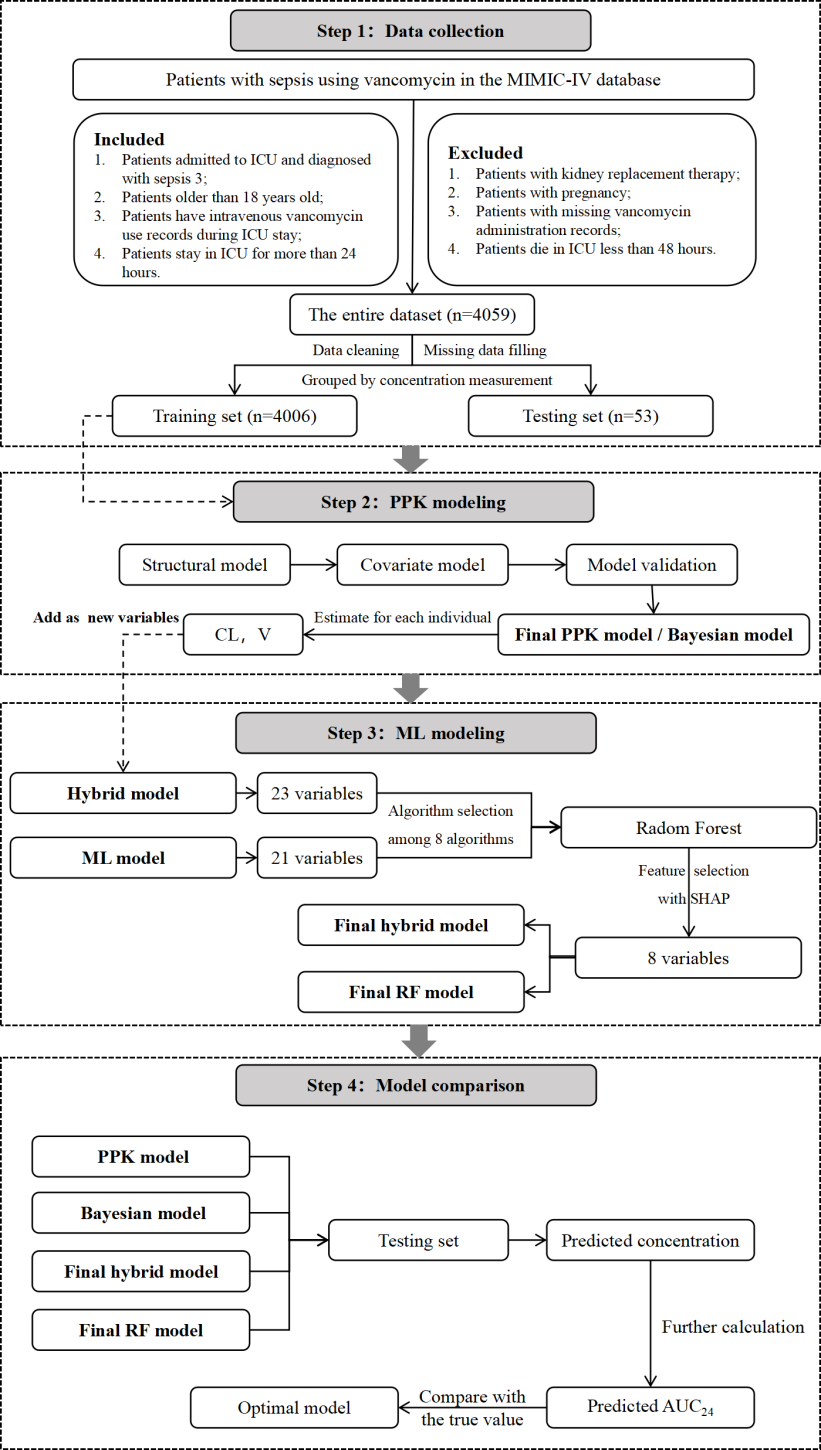
The workflow of data processing and algorithm selection.

The predicted AUC_24_ values from each model were compared with the true AUC_24_ values. MAE, MSE, RMSE, R², MAPE, and the percentage of prediction errors within ±30% (F30) were calculated to assess the predictive performance of each model. The workflow of data processing, algorithm selection, and modeling is illustrated in Fig. 1.

The MAE is the average of the absolute value of the deviation between the observed and predicted values. The mean of MSE deviation squared quantifies the overall degree of change in prediction error. RMSE is the square root of MSE and highlights the absolute magnitude of the error. MAPE is the normalized form of MAE and measures the proportion of error relative to the actual value. The value range of R^2^ is [0,1]; the larger the value, the better the model fitting effect. The proportion of predicted values with an error within 30% in F30 was used to measure the distribution of model prediction accuracy. The calculation equations for the above indicators are all shown below (2, 7–13), where ${\hat{y}}_{i}$is the predicted value of the ith patient, $y_{i}$ is the true value of the ith patient, and $\overset{-}{y}$ is the average true value of all patients.


(7) $$MAE=\frac{1}{n}\sum_{i=1}^{n}|\hat{y_{i}}-y_{i}|$$

(8) $$MSE=\frac{1}{n}\sum_{i=1}^{n}(\hat{y_{i}}-y_{i}{)}^{2}$$

(9) $$RMSE=\sqrt{\frac{1}{n}\sum_{i=1}^{n}(\hat{y_{i}}-y_{i}{)}^{2}}$$

(10) $$MAPE=\frac{100\%}{n}\sum_{i=1}^{n}|\hat{y_{i}}-y_{i}|$$

(11) $$SSR=\sum_{i=1}^{n}({\hat{y}}_{i}-\bar{y}{)}^{2}$$

(12) $$SSE=\sum_{i=1}^{n}(y_{i}-\hat{y_{l}}{)}^{2}$$

(13) $$\begin{array}{c} SST=SSR+SSE=\sum_{i=1}^{n}{\left(y_{i}-\overset{-}{y}\right)}^{2} \end{array}$$

(14) $$\begin{array}{c} R^{2}=1-\frac{SSE}{SST} \end{array}$$

## RESULTS

### Data collection

A total of 4,059 eligible patients were collected from the MIMIC-IV database, 53 patients with C_max_ and C_min_ measurement points in the same dosing cycle were used as the testing set, and the remaining 4,006 patients were used as the training set. The specific characteristics of the patients in the two groups are shown in Table 1. The average age of the patients was 65.3 years, and the overall average SOFA score was 6.21. The SOFA score in the training set was significantly higher than that in the testing set (6.22 vs 5.36, *P* <0.05). The average length of hospital stay of the patients in the testing set was significantly longer than that in the training set (22.3 days vs 15.5 days, *P* <0.001).

**TABLE 1:** The clinical characteristics of the patients^*a*^

Factor	Overall (*N* = 4,059)	Train set (*N* = 4,006)	Test set (*N* = 53)	*P* value
Demographic variables				
Age (SD)	65.39 (15.81)	65.41 (15.82)	64.17 (14.84)	0.479
Weight (SD)	82.56 (25.35)	82.49 (25.34)	87.49 (26.05)	0.182
Male (%)	2,387 (58.81%)	2,353 (58.74%)	34 (64.15%)	0.512
Race (%)				
Asian (%)	109 (2.69%)	109 (2.72%)	0 (0.00%)	0.403
White (%)	2,651 (65.31%)	2,619 (65.38%)	32 (60.38%)	0.469
Black (%)	442 (10.89%)	434 (10.83%)	8 (15.09%)	0.370
Other (%)	857 (21.11%)	844 (21.07%)	13 (24.53%)	0.502
Laboratory variables				
Creatinine (SD)	1.36 (1.27)	1.36 (1.28)	1.06 (0.50)	0.372
Clcr (SD)	93.25 (66.15)	93.28 (66.42)	91.19 (41.74)	0.357
Lactate (SD)	2.16 (1.47)	2.16 (1.48)	1.83 (0.87)	0.234
Hematocrit (SD)	29.57 (5.17)	29.59 (5.17)	28.53 (5.08)	0.116
White blood cell (SD)	13.68 (9.29)	13.65 (9.13)	15.85 (17.74)	0.352
Red blood cell (SD)	3.24 (0.62)	3.25 (0.62)	3.12 (0.62)	0.172
Hemoglobin (SD)	9.51 (1.73)	9.51 (1.73)	9.18 (1.76)	0.112
Platelet (SD)	215.36 (117.81)	215.24 (117.64)	224.41 (130.48)	0.567
Prothrombin time (SD)	17.47 (8.72)	17.49 (8.76)	15.99 (4.34)	0.856
Severity scores and indices				
SOFA (SD)	**6.21** (**3.43**)	**6.22** (**3.43**)	**5.36** (**3.35**)	**<0.05^*b*^**
CCI (SD)	5.63 (3.10)	5.62 (3.10)	5.91 (2.84)	0.291
APSIII (SD)	51.07 (19.47)	51.09 (19.51)	49.13 (16.40)	0.593
SQPSII (SD)	40.17 (13.43)	40.21 (13.44)	37.81 (12.73)	0.167
GCS (SD)	13.38 (2.88)	13.38 (2.88)	13.57 (2.19)	0.458
Clinical outcomes				
ICU_LOS (SD)	7.17 (7.60)	7.15 (7.58)	8.37 (9.01)	0.604
HOS_LOS (SD)	**15.62** (**13.09**)	**15.54** (**12.99**)	**22.32** (**17.81**)	**<0.001^*b*^**
ICU_ Death (%)	484 (11.92%)	478 (11.93%)	6 (11.32%)	1
HOS_ Death (%)	750 (18.48%)	743 (18.55%)	7 (13.21%)	0.414

^*a*^ Clcr, creatinine clearance; CCI, Charlson Comorbidity Index; GCS, Glasgow Coma Scale; ICU_LOS, intensive care unit length of stay; HOS_LOS, hospital length of stay.

^*b*^ Statistically significant differences (p < 0.05) between the train and test sets.

### PPK model

Based on 11,046 drug concentration data of 4,006 patients in the training set, a one-compartment model with first-order absorption was used to describe the concentration and time distribution of vancomycin. The exponential model described the interindividual variation in equation 1 and the hybrid model described the residual variation in equation 4. The final PPK model is as follows:


(15) $$\begin{array}{c} CL=3.35\times (CLCR/93{)}^{0.997}\times e^{-0.151\times (CCI/5.62)}\times e^{{ŋ}_{1}} \end{array}$$

(16) $$\begin{array}{c} V=98.5\times (WT/84{)}^{0.205}\times e^{{ŋ}_{2}} \end{array}$$

In the equation, CLCR represents creatinine clearance, CCI represents Charlson Comorbidity Index score, and WT represents patient weight in kilograms. ŋ_1_ and ŋ_2_ denote interpatient variation. The typical value of the CL population was 3.35 mL/min, and the typical value of the V population was 98.5 L.

Figure S1 shows that the PPK model demonstrates both high prediction accuracy and low bias. The bootstrap parameter results showed that the PPK parameter estimates all fell within the 95% confidence interval, as shown in Table S1. The above results showed that the prediction ability of the model was good and the parameter estimates were reliable.

### Machine learning model

The benchmarking results for the ML and hybrid models are presented in Fig. S2, S3 and Table S2 and S3. The results indicate that the Random Forest model demonstrated the best predictive performance among both ML model and hybrid model. For the ML model, the Random Forest achieved an R² of 0.2, MAE of 0.66, RMSE of 0.89, and MSE of 0.79. For the hybrid model, it achieved an R² of 0.4, MAE of 0.57, RMSE of 0.76, and MSE of 0.58. Consequently, Random Forest was selected as the ML algorithm for this study.

**Fig 2:**
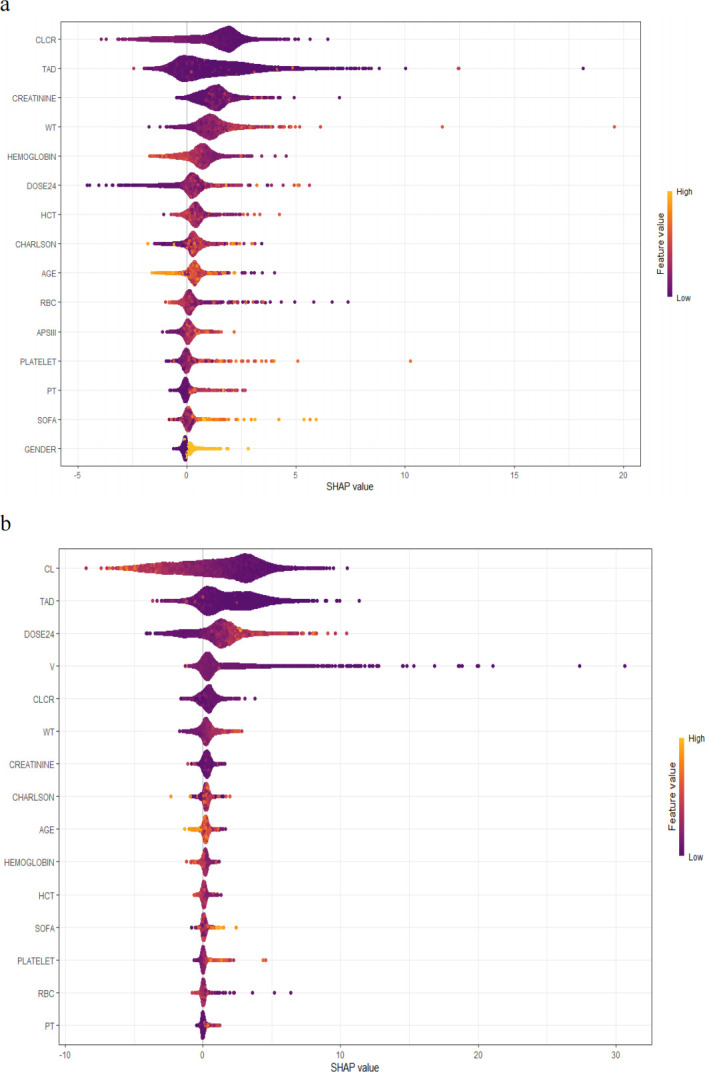
Variable selection SHAP plot. (a) SHAP values for the Random Forest model. (b) SHAP values for the hybrid model. The vertical axis represents the feature names, sorted by importance from high to low, with the most important feature at the top. The horizontal axis represents the SHAP values, indicating the impact of each feature on the model’s predictions. Positive values indicate that the feature increases the model’s prediction, while negative values indicate that the feature decreases the prediction. The width of each feature represents the distribution of SHAP values across different samples; wider areas suggest that the feature’s contribution varies more across samples. The color represents the magnitude of the feature values, with purple indicating lower values and yellow indicating higher values.

The Boruta method showed that all variables were included, and the SHAP method obtained the contribution of each feature to the prediction result, and the specific results are shown in Fig. 2. In the Random Forest model, the most important variable was CLCR, followed by time after dose (TAD). The most important variable for the hybrid model was CL, followed by TAD. TAD, a 24 hour dose of vancomycin (DOSE24), weight (WT), age, hematocrit (HCT), red blood cell count, hemoglobin, and CLCR were selected as modeling variables in the final Random Forest model. TAD, DOSE24, CL, V, age, WT, HCT, and hemoglobin were selected as modeling variables for the hybrid model. On this basis, hyperparameter tuning was performed to obtain the final model. After tuning, the R^2^ of the simple Random Forest model increased from 0.2 to 0.6, and the R^2^ of the hybrid model increased from 0.4 to 0.7.

### Model comparison

**Fig 3:**
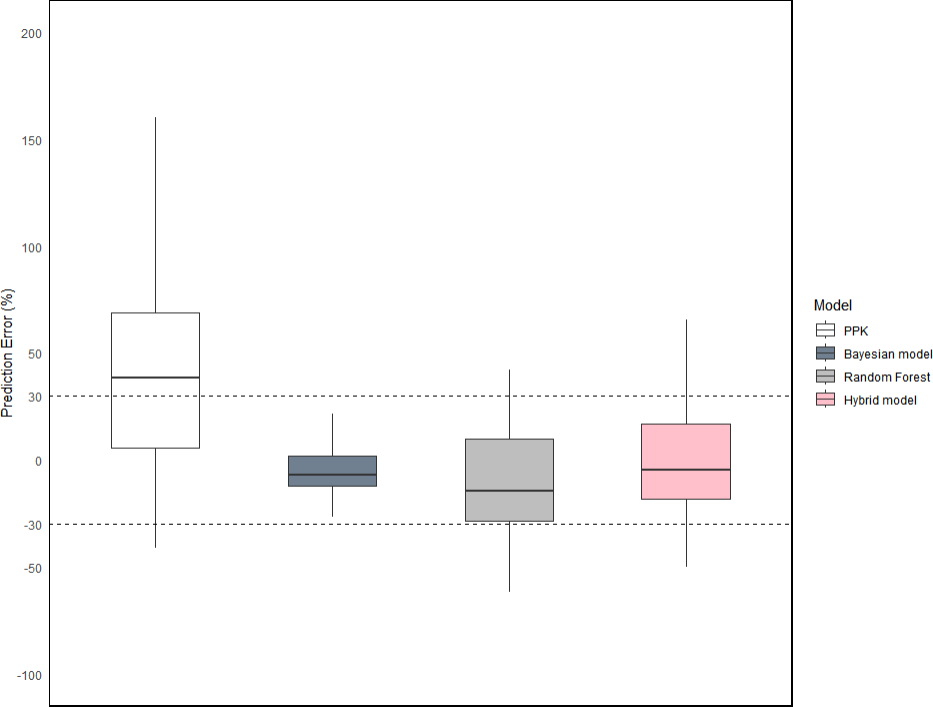
Boxplot of F30 for each model. Each box represents a model, and the vertical axis represents the percentage of prediction errors. Two dashed lines indicate the ±30% error range as a reference benchmark. The closer the data points are concentrated within this range, the better the model’s performance.

**Fig 4:**
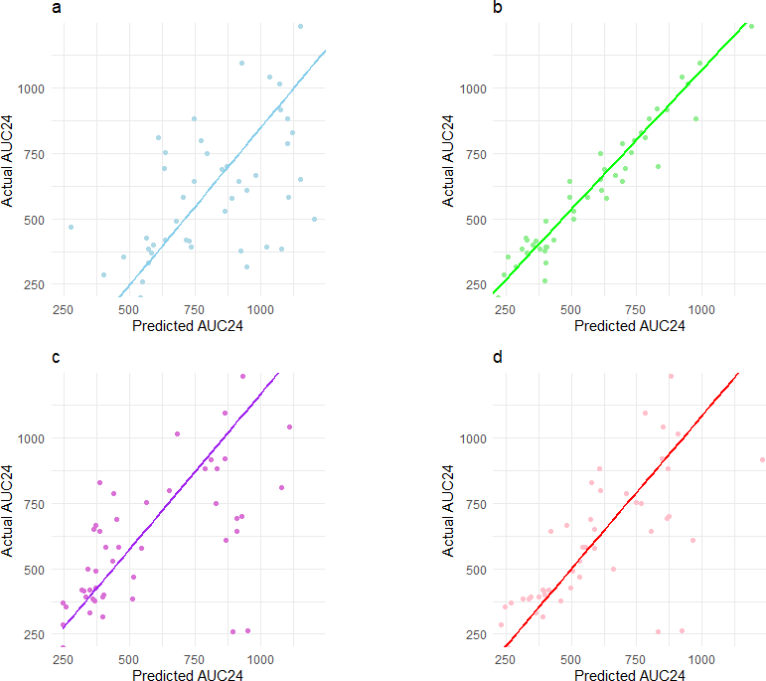
Model fit plots. (a) PPK model predicted AUC_24_ vs actual AUC_24_. (b) Bayesian model predicted AUC_24_ vs actual AUC_24_. (c) Random Forest model predicted AUC_24_ vs actual AUC_24_. (d) Hybrid model predicted AUC_24_ vs actual AUC_24_. The horizontal axis represents the predicted AUC_24_ values from the models, and the vertical axis represents the actual AUC_24_ values. The regression line in each plot shows the trend relationship between the model’s predictions and the actual values.

This study developed four models: the PPK model, Bayesian model, Random Forest model, and hybrid model. The comparison of AUC_24_ predictions from each model in the testing set is summarized in Table 2. Among them, the Bayesian model demonstrated the smallest error, offering superior accuracy and stability, followed by the hybrid and Random Forest models. The PPK model exhibited the largest prediction error. Figure 3 and 4 illustrate that the Bayesian, hybrid, and Random Forest models showed relatively small prediction errors, whereas the PPK model displayed substantial errors.

**TABLE 2:** Model prediction results in the testing set

Group	MAE	MSE	RMSE	MAPE (%)	R^2^	F30 (%)
PPK model	308.91	169162	308.91	68.17	0.64	34.6
Bayesian model	84.91	15177	84.94	13.37	0.968	94.2
Random Forest	231.99	170093	231.99	34.17	0.559	61.5
Hybrid model	179.32	111204	179.32	28.52	0.703	76.9

When vancomycin concentration was available, the Bayesian model outperformed the others, with an F30 of 94.2% and MAPE of 13.37%. In the absence of vancomycin concentration, the hybrid model provided the best predictions, achieving an F30 of 76.9% and MAPE of 28.52%, followed by the Random Forest model with an F30 of 61.5% and MAPE of 34.71%. The PPK model exhibited the poorest prediction performance, with an F30 of 34.6% and MAPE of 68.17%.

## DISCUSSION

This study developed four predictive models for sepsis patients receiving vancomycin treatment: the PPK model, Bayesian model, ML model, and hybrid model. When vancomycin concentration data were unavailable, the hybrid model demonstrated the best performance in terms of AUC_24_ prediction, with a lower MAE and a higher R² value compared to the other models. Meanwhile, when vancomycin concentration data were available, the Bayesian model exhibited the highest predictive accuracy, with the lowest MAE and highest R² value.

The PPK model showed relatively poorer performance in comparison. This can be attributed to its reliance on simplified pharmacokinetic assumptions and the limited ability to account for patient-specific variables that influence drug pharmacokinetics. Additionally, the sparse sampling of patient (4,006/4,059) data in the MIMIC-IV database further limited the model’s ability to accurately capture the variability in vancomycin exposure. The higher MAE and lower R² values suggested that the PPK model was less effective in capturing the variability in vancomycin exposure within the patient population of this study.

ML is increasingly being adopted in the medical field, leveraging the vast clinical data and knowledge recorded in electronic health records to train algorithms. Previous studies have highlighted the advantages of ML, including improved accuracy, robustness, reduced overfitting, and an enhanced ability to capture complex features and nonlinear relationships (22). In the context of individualized precision treatment, ML employs data analysis and predictive modeling to guide more accurate diagnoses, tailored treatments, and outcome predictions (23). In this study, the Random Forest model demonstrated the best predictive performance among the ML models. This finding is consistent with previous research on drug concentration prediction (24), suggesting that Random Forest excels in managing complex clinical data and capturing nonlinear relationships among variables (25).

In our study, pharmacokinetic (PK) parameters were incorporated as variables into the ML model to construct a hybrid model. Compared with the PPK model and the ML model without PK parameters, the inclusion of PK parameters significantly enhanced the model’s predictive performance, yielding MAPE improvements of 58% and 17%, respectively. This underscores the advantages of combining ML with PPK. Our findings suggest that this integration holds significant promise in improving prediction accuracy and optimizing individualized treatment for patients.

This study proposes two model selection strategies for predicting AUC_24_ under different clinical scenarios. When patient vancomycin concentrations are accessible or can be measured, the Bayesian model is a suitable option. This is because the Bayesian model integrates prior concentration data with current measurements to predict posterior concentrations, resulting in predictions that more closely align with actual values, thus improving AUC_24_ estimation accuracy. Although the Bayesian model outperforms the other models in terms of overall performance, its practical application is contingent upon the availability of drug concentration data, which may be restricted in clinical settings. This highlights the importance of considering the feasibility of clinical application, particularly in fast-paced decision-making scenarios. Consequently, in situations where drug concentration data is not available, the hybrid model remains the optimal choice, offering greater adaptability to diverse and complex clinical conditions.

The strengths of this study lie primarily in the enhancement of the PPK model through the integration of ML methods, which significantly improves predictive performance and facilitates AUC_24_ prediction. Furthermore, this study offers a comprehensive comparison of multiple models. While most similar studies have either relied solely on ML models to predict antibiotic exposure (26, 27), or performed pairwise comparisons between the hybrid and PPK models using simulated data (28), our study and the research mentioned above exhibit several key differences. First, the objectives of the studies differ. While our study focuses on predicting the AUC, the other studies concentrate on aspects such as the clearance rate. Second, the data sources used in the studies vary. Our study utilizes real-world data from the MIMIC-IV database, whereas some of the other studies rely on simulated patient data. Additionally, the approach to variable selection differs. Li et al.’s study (28) used a limited set of variables, including sex, age, weight, and creatinine, while our study considered over 20 variables, including CL and V, and selected the eight most relevant ones for AUC prediction. Finally, our study constructs and compares four distinct models, including a hybrid model, to predict the AUC_24_ of vancomycin under various conditions. By comparing all models, we aim to identify the optimal model for different clinical scenarios, thereby providing guidance for clinicians in the more rational use of vancomycin in sepsis patients.

Our study has several limitations. First, the data used is derived from the MIMIC-IV database, a single-center source, which may limit the generalizability of the model. Moreover, the drug concentration data for many patients in this database are sparsely sampled, which does not fully capture the distribution, metabolism, and excretion processes of vancomycin, potentially introducing bias into the established PPK model. Second, while we evaluated eight ML algorithms for comparative analysis and ultimately chose the Random Forest model, other algorithms that were not considered may also have potential. Third, some physiological and biochemical indicators important for prediction may have been overlooked. Fourth, due to missing height data in the MIMIC database, BMI could not be calculated for 61.7% of patients, preventing the incorporation of adjusted weight for obese patients in these models. Finally, the approach of incorporating PK parameters as key variables in the ML model to construct a hybrid model warrants further exploration to determine if it represents the optimal approach.

### Conclusion

In conclusion, this study successfully established four models for predicting the AUC_24_ of vancomycin in patients with sepsis. In the absence of concentration data, the hybrid model outperformed other models, offering superior accuracy. When the vancomycin concentration was available, the Bayesian model demonstrated the highest predictive performance. This research provides valuable insights for clinicians managing vancomycin therapy in patients with severe sepsis and introduces a novel approach that merges machine learning algorithms with pharmacometrics. The findings of this study may also have broader applications for predicting the exposure of other medications.

## SUPPLEMENTAL MATERIAL

Supplemental material spectrum.00499-25-s0001.pdf Fig. S1 to S3; Tables S1 to S3.

ASM does not own the copyrights to Supplemental Material that may be linked to, or accessed through, an article. The authors have granted ASM a non-exclusive, world-wide license to publish the Supplemental Material files. Please contact the corresponding author directly for reuse.
