# Supplementary material for: Machine learning and population pharmacokinetics: a hybrid approach for optimizing vancomycin therapy in sepsis patients
Source: Microbiol Spectr. 2025 Mar 31;13(5):e00499-25. doi: 10.1128/spectrum.00499-25 (PMC12054080; doi:10.1128/spectrum.00499-25)

Figure Legends

Figure S1 Goodness-of-fit (GOF) of the PPK model

a. Individual predictions versus observed concentrations.

b. Population predictions versus observed concentrations.

c. Conditional weighted residuals versus population predictions (CWRE-PRED) plot. The x-axis represents the population predicted concentration, and the y-axis represents the conditional weighted residuals. This plot measures the deviation between the model's predictions and the observed values.

d. Conditional weighted residuals versus time (CWRE-TIME) plot. The x-axis represents the time elapsed after dosing, and the y-axis represents the conditional weighted residuals. This plot measures the deviation between the model's predictions and the observed values.

Figure S2 Boxplot of Baseline Performance Results for the Machine Learning Model

a. Boxplot of MAE for machine learning model.

b. Boxplot of RMSE for machine learning model.

c. Boxplot of MSE for machine learning model.

d. Boxplot of R^2 for machine learning model.

Figure S3 Boxplot of Baseline Performance Results for the Hybrid Model

a. Boxplot of MAE for hybrid model.

b. Boxplot of RMSE for hybrid model.

c. Boxplot of MSE for hybrid model.

d. Boxplot of R^2 for hybrid model.

Table S1 Bootsrap Result

Factor	Parameter estimation	Bootstrap		
	Estimate （RSE%）	Median	95% Confidence Interval	Bias
CL（L·h^{-1}）	3.35（3%）	3.33	3.15-3.54	-1.0%
CLCR on CL	0.997（2%）	0.99	0.94-1.04	-0.6%
Charlson on CL	-0.151（17%）	-0.15	-0.21--0.09	-0.2%
V（L）	98.5（1%）	98.80	95.46-101.59	2.7%
WT on CL	0.205（23%）	0.20	0.11-0.30	-0.1%

Table S2 Baseline Performance Table of the Machine Learning Model

Model	MAE	RMSE	MSE	R^2
GLM with Elastic Net Regularization	0.74	0.98	0.96	0.01
k-Nearest Neighbors Regression	0.71	0.97	0.93	0.07
Linear Model	0.73	0.97	0.94	0.04
Neural Network	0.71	0.93	0.87	0.1
Random Forest	0.66	0.89	0.79	0.2
Decision Tree	0.73	0.97	0.93	0.03
Support Vector Machine	0.68	0.92	0.85	0.13
XGBoost	0.81	1.03	1.05	-0.07

Table S3 Baseline Performance Table of the Hybrid Model

Model	MAE	RMSE	MSE	R^2
GLM with Elastic Net Regularization	0.67	0.89	0.79	0.18
k-Nearest Neighbors Regression	0.68	0.91	0.83	0.77
Linear Model	0.66	0.88	0.77	0.21
Neural Network	0.61	0.81	0.66	0.34
Random Forest	0.57	0.76	0.58	0.4
Decision Tree	0.65	0.85	0.73	0.24
Support Vector Machine	0.59	0.8	0.63	0.35
XGBoost	0.76	0.96	0.93	0.03

Figure S1 GOF of PPK model

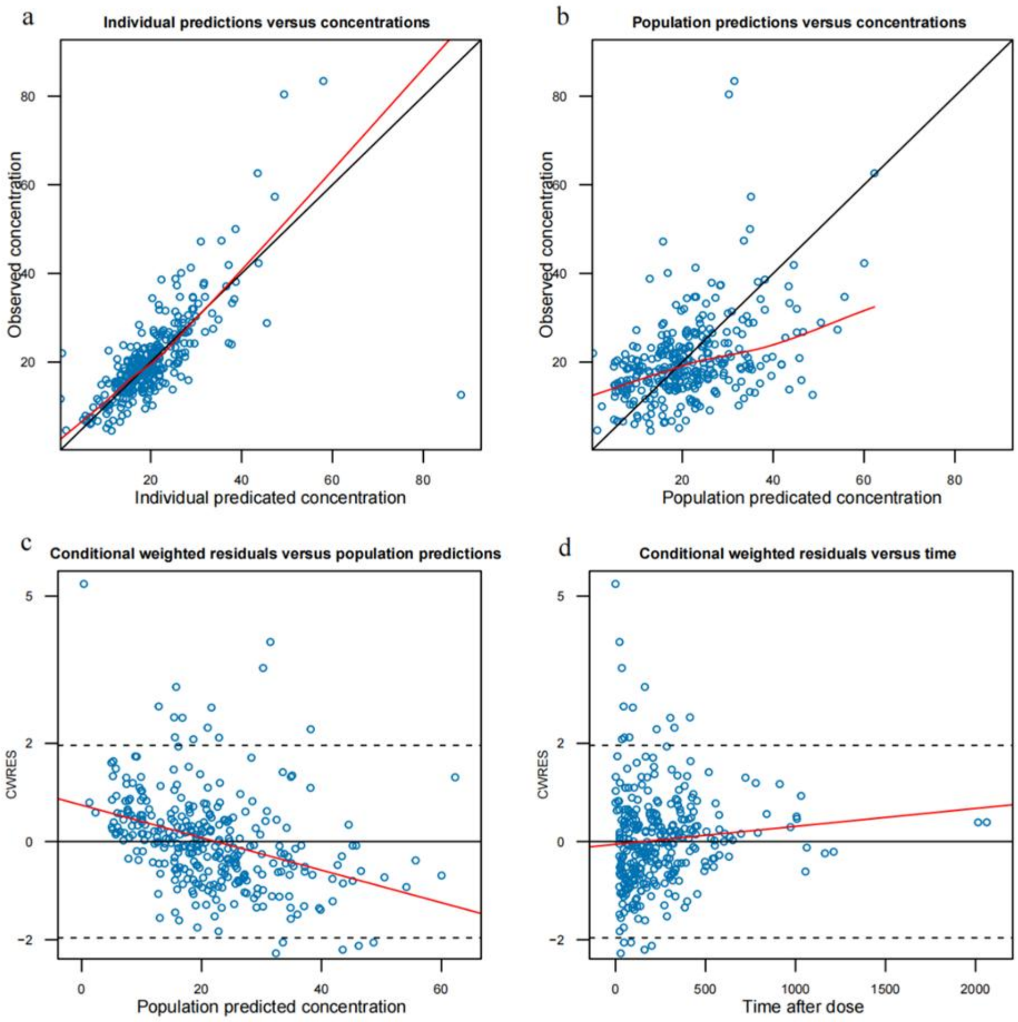

Figure S2 Boxplot of Baseline Performance Results for the Machine Learning Model

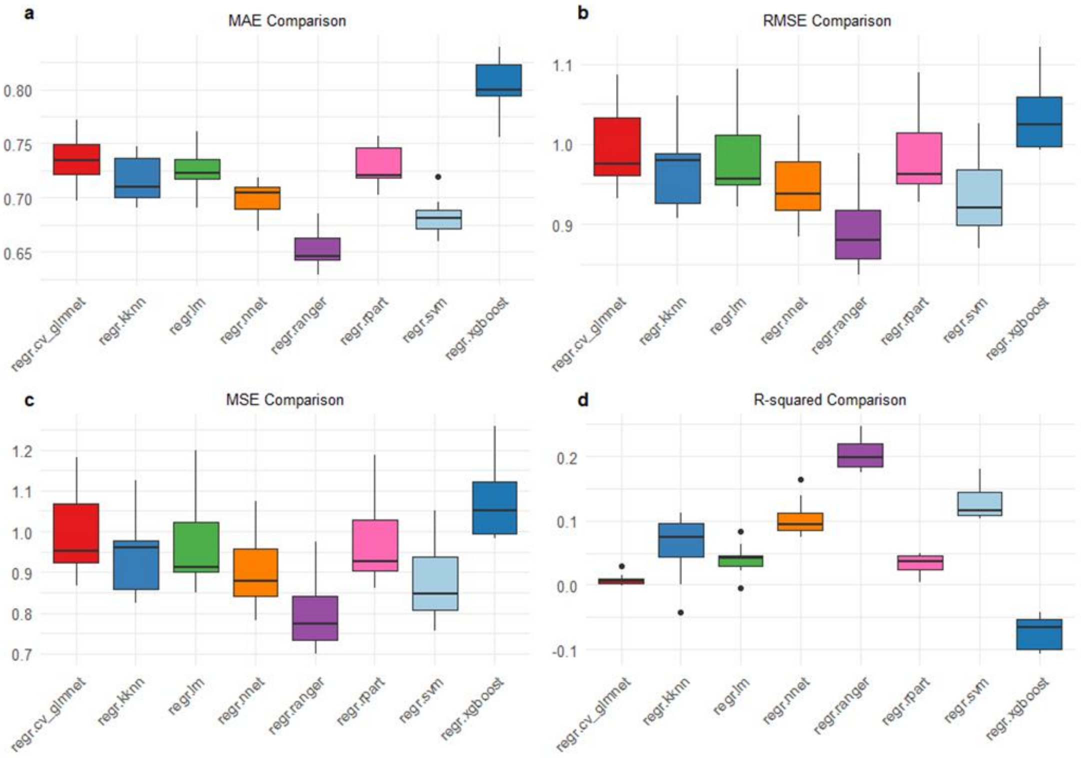

Figure S3 Boxplot of Baseline Performance Results for the Hybrid Model

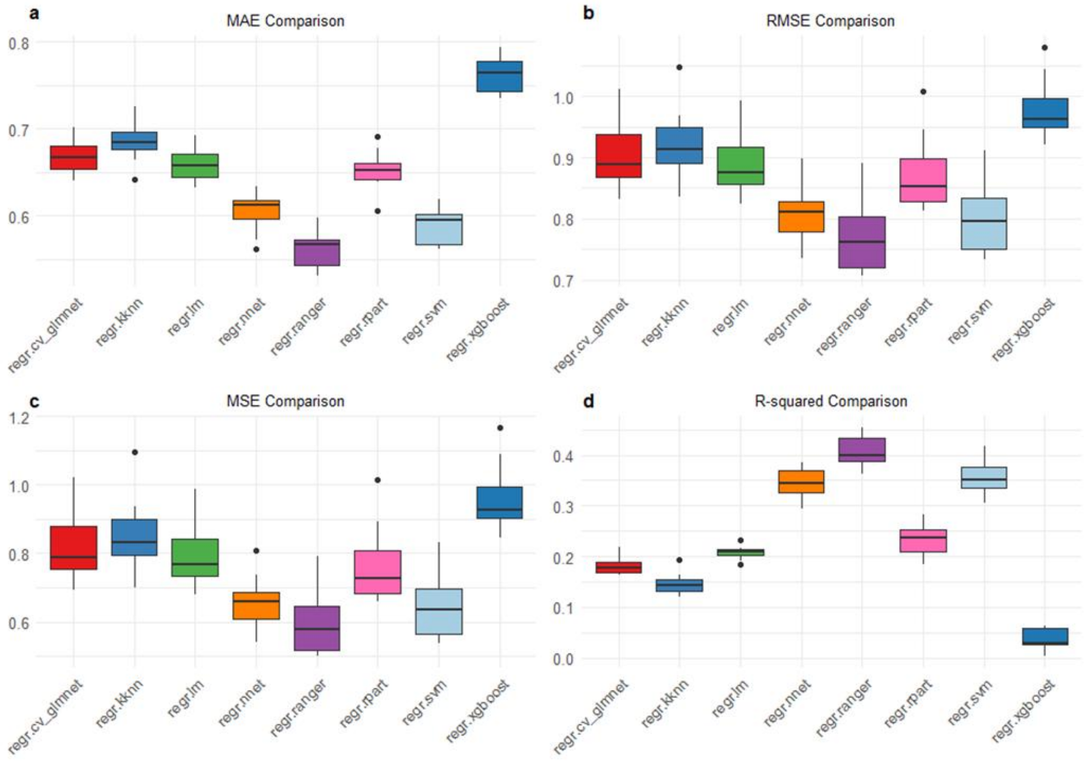

Supplement: Supplemental material — Fig. S1 to S3; Tables S1 to S3. [file spectrum.00499-25-s0001.pdf]
